# Minimum acceptable diet and associated factors among children aged 6–23 months in Ethiopia

**DOI:** 10.1186/s13052-021-01169-3

**Published:** 2021-10-30

**Authors:** Haimanot Abebe, Molla Gashu, Aynalem Kebede, Habtemariam Abata, Alex Yeshaneh, Haile Workye, Daniel Adane

**Affiliations:** 1 Department of Public Health, College of Medicine and Health Sciences, Wolkite University, Wolkite, Ethiopia; 2 Yeka sub-city health offices, Addis Ababa, Ethiopia; 3 Ethiopian Federal food, medicine, and health care administration and control authority offices, Addis Ababa, Ethiopia; 4 Department of Midwifery, College of Medicine and Health Sciences, Wolkite University, Wolkite, Ethiopia; 5 Department of Nursing, College of Medicine and Health Sciences, Wolkite University, Wolkite, Ethiopia

**Keywords:** Dietary diversity, Meal frequency, Minimum acceptable diet, Addis Ababa, Ethiopia

## Abstract

**Introduction:**

The health and growth of children less than two years of age can be affected by the poor quality of complementary foods and poor feeding practices even with optimal breastfeeding. In Ethiopia, empirical evidence on the minimum acceptable diet and its associated factors is limited. Therefore, this study was aimed to assess the level of minimum acceptable diet and its associated factors among children aged 6–23 months in Addis Ababa Ethiopia.

**Methods:**

An institution-based Cross-sectional study was conducted among a total of 575 mother-child pairs. A simple random sampling technique was used to recruit participants. For infant and young child feeding practices, the data collection tools were adapted from world health organizations’ standardized questionnaire which is developed in 2007. Data entry and analysis were performed using EPI data version 3.1 and SPSS version 20 respectively. Bivariable and multivariable logistic regression analyses were performed to determine predictor variables. Statistical significance was declared at *p*-value < 0.05.

**Result:**

In this study, the level of minimum acceptable diet was found to be 74.6%.. About 90.6 and 80.2% of the children received minimum meal frequency and dietary diversity respectively. Having a husband secondary and above educational level [AOR = 4.789(95%CI:1.917–11.967)], being a housewife [AOR = 0.351(95% CI: 0.150–0.819)], having a history of more than three postnatal follow-ups [AOR = 2.616(95%CI:1.120–6.111], Having mothers age between 25 and 34 years [AOR = 2.051(95%CI:1.267–3.320)], being male child [AOR = 1.585(95%CI:1.052–2.388)] and having children age between 18 and 23 months [AOR = 3.026(95%CI:1.786–5.128)] were some of the factors significantly associated with a minimum acceptable diet.

**Conclusion:**

In this study, the minimum acceptable diet among children aged 6–23 months was significantly associated with the educational status of the husband, mother’s occupation, history of postnatal follow-up, age of the mother, sex of the child, and age of the child. Thus, attention should be given to educating the father, empowering mothers to have a job, promoting gender equality of feeding, and counseling on the benefit of postnatal care visits. In addition, the ministry of health should work on educating and advocating the benefit of feeding the recommended minimum acceptable diet to break the intergenerational cycle of malnutrition.

## Introduction

Improving nutrition through appropriate complementary feeding practices is crucial for the achievement of healthy growth, development, and survival of young children and is one of the most cost-effective approaches to address many of the societal, environmental, and economic challenges worldwide [1–3]. The World Health Organization (WHO) recommends the indicators of proper complementary feeding as starting solid, semi-solid, or soft foods, minimum meal frequency, minimum dietary diversity, minimum acceptable diet, and consumption of iron-rich or iron-fortified foods [4].

Insufficient quantities and inadequate quality of complementary foods, poor child feeding practices, and high rates of infections harm health and growth in children less than 2 years of age [5]. Any damage caused by nutritional deficiencies during the first two years of life can lead to impaired cognitive development, compromised educational achievement, and low economic productivity [6].

Globally, ensuring optimal complementary feeding can avert a substantial proportion of childhood deaths [5]. Worldwide, poor Infant and Young Child Feeding practices have been identified as significant contributors to under-nutrition. South Asia is the hardest hit of childhood under-nutrition worldwide [7, 8]. Sub-Saharan African countries carry the highest risk of this global burden. In Ethiopia, only 8% of infants aged 6–23 months receive complementary foods while continuing to be breastfed [9]. According to the National demographic health survey of Ethiopia (EDHS) 2016, the prevalence of minimum dietary diversity (MDD), meal frequency, and minimum acceptable diet (MAD) was 14, 45, and 7% respectively [9]. Several studies in Ethiopia showed that the minimum acceptable diet in children whose age between 6 and 23 months was 8.6% in Dembecha, 6.1% in other parts of Ethiopia, 8.8% in two agro-ecological zones of rural Ethiopia, 21.1% in Wolaita Sodo, and 8.4% in Gorche District [10–14]. Similar studies in Addis Ababa also revealed that the prevalence of minimum meal frequency (MMF), MDD, and MAD was 67.4, 43.4, and 27.1% respectively [9].

In many low-income countries including Ethiopia, meeting MDD and MMF standards has been a serious challenge, especially in areas where household food security is poor [15]. Culture affects the practice of dietary diversity, meal frequency, and minimum acceptable diet [16]. Despite increased attention to undernutrition; nutritional deficiencies remain a multifaceted problem affecting infants, young children, adolescent girls and women. Information on dietary diversity, meal frequency minimum acceptable diet, and associated factors are needed to prioritize, design and initiate further intervention programs aimed at improving dietary diversity and meal frequency to reduce under nutrition in children [17].

In Ethiopia, considerable progress **has been made in the development of complementary feeding and the provision of national nutrition programs** by defining standards for appropriate feeding through the notable publication of different guiding principles. However, the strategies for breaking the inter-generational cycle of malnutrition were not well focused and not complete, particularly in addressing the critical window of opportunities “the first 1000 days” and critical elements of efforts such as encouraging and supporting appropriate complementary feeding for children aged 6–23 months [18].

Empirical evidence on minimally acceptable diet and associated factors are limited in Ethiopia and they are in the rural communities and district level [13]. Therefore, this study was aimed to assess the prevalence of a minimum acceptable diet and its associated factors among children aged 6–23 months in the capital city of Ethiopia. So, the findings of this study will help the Ministry of health and its partners who are working on nutrition-related activities to prioritize the problem design and initiate further intervention programs.

## Methods and materials

### Study design and settings

An institution-based cross-sectional study was conducted from June 01 to June 30, 2019, in the city of Addis Ababa Ethiopia. The city comprises 10 sub-cities (Kifle Ketemas). Yeka sub-city, Bole sub-city, and Arada sub-city were three of the ten sub-cities with a total population of 454,850, 406,059, and 279,020 respectively. The expected number of children aged 6–23 months was 21,872 (8719 from yeka sub-city, 7796 from Bole sub-city and 5357 from Arada sub-city). Yeka sub-city had 14 districts and 15 health centers and one governmental hospital, Bole sub-city had 10 health centers and 15 districts and Arada sub-city had 9 health centers and 10 districts (Addis Ababa city administration health bureau of 2011 E. C data).

### Populations

All children aged 6–23 months who came for the expanded program on immunization (EPI) at the government health facility in Addis Ababa, Ethiopia were the source population whereas those children aged 6–23 months and came to the Expanded Program on Immunization (EPI) during the data collection period at the selected governmental health centers were taken as the study population.

### Eligibility criteria

All children aged 6–23 months with a permanent residence of the mother (lived for at least six months) who came for the Expanded Program on Immunization (EPI) only. Children aged 6–23 months whose mothers had (permanent residents) in the study area during data collection. While those children aged 6–23 months with known medical or surgical problems were excluded.

### Operational definition

Minimum dietary diversity: was taken as an achieved if the children were received four or more food groups from of the seven food groups such as grains, roots and tubers; legumes and nuts; dairy products (milk, yogurt); Flesh foods (meat, fish, poultry, and liver/organ meats); eggs; vitamin A-rich fruits and vegetables; and other fruits and vegetables [19–21].

Minimum meal frequency: The children who received solid, semisolid, or soft foods is taken as minimal meal frequency and was measured when the infant feeds twice for breastfed infants 6–8 months, three times for breastfed children 9–23.9 months, and four times for non-breastfed children 6–23 months [19, 21].

Minimum acceptable diet: For breastfed children, it is achieved if the child meets both the MDD and MMF criteria. For non- breastfeed children, the child has to receive at least four food groups excluding dairy products, two milk feeds, and MMF [19].

Satisfactory exposure to media: If Women aged 15–49 years read a newspaper or magazine or listen to the radio, or watched television at least once a week [15].

Household income: consists of all receipts whether monetary or in-kind (goods and services) that are received by the household or by individual members of the household at annual or more frequent intervals, but exclude windfall gains and other such irregular and typically one-time receipts [22].

Household food security: households who experience none of the food insecurity (access) conditions, or just experience worry, but (one or two times in the last 4 weeks) are labeled as “Food secured” [23, 24].

Household food insecure: in the ability of households to access sufficient food at all time to lead to an active healthy life (includes all stage of food insecurity; mild, moderate and severe) without eating), even as infrequently as rarely (one or two times in the last 4 weeks) [25].

Maternal decision making: if the mother has the right to decide on the amount of food type of food and the right to buy food for the baby, then the mother is said to be involved in the decision making. However, if she doesn’t involve in any of the above criteria, then the mother is said to be not involved in the decision-making.

Timely introduction of complementary feeding: the Introduction of solid, semi-solid, or soft foods, minimum meal frequency, minimum dietary diversity, and consumption of iron-rich or iron-fortified foods and started at six months of age [21, 26].

Appropriate:-if the mother responds correctly to all four indicators (timely introduction of complementary feeding, MMF, MDD, and MAD).

Inappropriate:-among the four indicators if at least one indicator was not fulfilled [27].

### Sample size determination

The minimum sample size was determined using a single and the double population proportion formula for the first and the second objectives respectively and was calculated using Epi Info™ version 7 stat calc. The final required sample size (large sample from the two objectives) for this particular study was obtained using the second objective and it was 575 [10].

### Sampling procedure

**Fig. 1:**
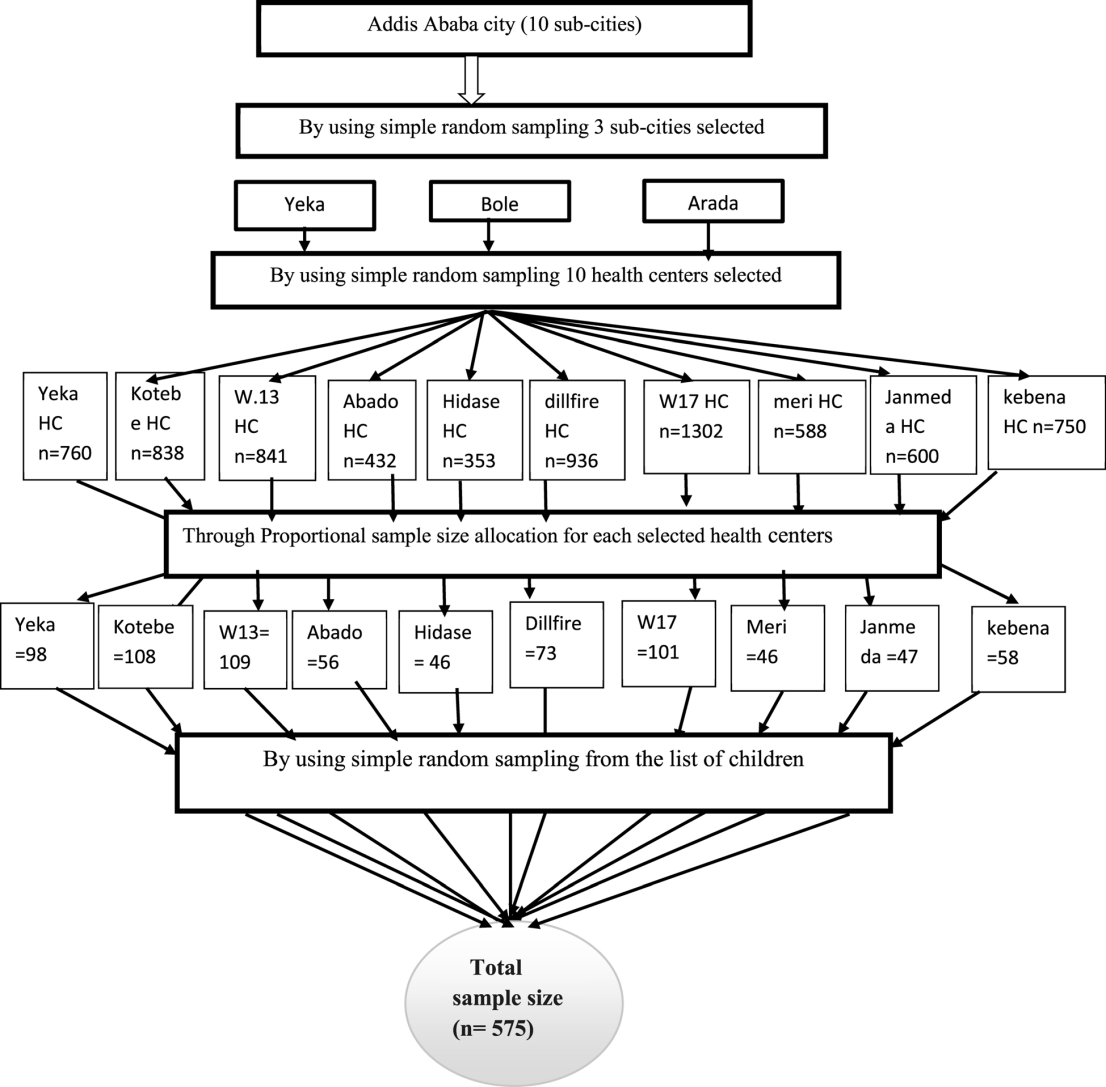
simple sketch map for sampling procedure of the study for minimum acceptable diet

Out of 10 sub-cities in the city, 30% of them (3 sub-cities such as Yeka, Bole and Arada) were selected by lottery method. From each sub-city 30% of their health centers (HC) were again selected by lottery method (from yeka 5 HC, from Bole 3 HC and Arada 2 HC). The eligible total number of children aged 6–23 months from each sub-city were selected using population size to proportional allocation based on their medical record number as sampling frame (Fig. 1).

### Data collection procedure and tool

A structured and pre-tested interviewer-administered questionnaire was prepared by reviewing relevant works of different literature. Primary data on the practice of minimum acceptable diet, minimum dietary diversity by 24 h method, minimum meal frequency, and related factors were collected from mothers or caregivers who had a child aged 6–23 months by using the 24-h recall method. Five experienced well-trained and experienced clinical nurses and two senior public health officers were recruited and trained for data collection and supervision, respectively. The data collection tool regarding the various factors is adapted from EDHS 2016 and different literature with some modifications to fit with the local context. Moreover, the tool on dietary diversity meal frequency was adapted from the WHO standardized questionnaire for IYCF practices [21].

### Data quality assurance

To ensure quality, the questionnaire was translated into the local language by experts. Finally, before data collection, it was re-translated back to English to verify consistency. Before starting the actual data collection, one day of extensive training was given for the data collectors and supervisors. A pre-test for appropriateness and feasibility of the tool was conducted and all necessary modifications and amendments were done accordingly. The tool was used with a reliability test or Cronbach’s alpha correlation coefficient of greater than or equal to 0.7 for inter-item consistency. The completeness and accuracy of questionnaires were checked daily before leaving the data collection site for immediate action. After data collection before analysis, all collected data were checked for completeness. Double data entry (data were entered by two people independently) was performed to check the consistency or reduce data entry error.

### Data processing and analysis

The collected data were coded, cleaned, edited, and entered into Epidata version 3.1 and exported to SPSS version 20.0 for statistical analysis. The presence of an association between explanatory and outcome variables was ascertained using binary logistic regression analysis. The goodness of fit was tested by the log-likelihood ratio (LR). To control all possible confounders all variables with *P* < 0.25 in the bivariate analysis were included in the final model of multivariable analysis. To see the correlation between independent variables multi-collinearity test was carried out by using collinearity statistics. Values with the standard error > 2 were dropped from the analysis. In a multivariable model adjusted odds ratio determined with a 95% confidence level was used to assess the strength of association. In this study *P*-value < 0.05 was deemed to declare statistical significance. Then, the finding was presented by using simple frequencies, summary measures, tables, texts, and figures.

## Results

### Socio-demographic characteristics of study participants

In this study, 562 respondents participated with a response rate of 97.7%). The median age of the mothers was 28 (SD ±6 years). More than half (50.7%) of the children were males. The majority (37.4%) of the children were in the age group of 18 and 23 months. The median age of the children was 16 (±4 years) months. All most all, (95.4%) of mothers were married, 406 (72.2%) and 339 (60.3%) of their husbands had a secondary and above level of education and were private workers respectively. Four hundred thirty-five (77.4%) were orthodox Christian religious followers. Four hundred six (72.2%) of fathers had an educational level of secondary and above, and 70.8% of the mothers had been working as a housewife **(See** Table 1**)**.

**Table 1:** Parental socio-demographic characteristics of children 6–23 months, Addis Ababa Ethiopia, 2019(*n* = 562)

Variables	Category	Frequency (%)
Maternal age group	15–24	112(19.9)
	25–34	386(68.7)
	35–49	64(11.4)
Sex of child	Male	285(50.7)
	Female	277(49.3)
Age of child	6–11 months	184(32.7)
	12–17 months	168(29.9)
	18–23 months	210(37.4)
Birth order of index child	First	278(49.5)
	2 and above	284(50.5)
Under-five children	one under 5 child	433(77.0)
	two & above child	129(23.0)
Maternal marital status	Currently not married	26(4.6)
	Married	536(95.4)
Ethnicity	Amhara	334(59.4)
	Oromo	81(14.4)
	Tigre	60(10.7)
	Others	87(15.5)
Religion	Orthodox	435(77.4)
	Protestant	45(8.2)
	Muslim	81(14.4)
Mother’s educational status	no formal education	44(7.8)
	primary education	203(36.1)
	2nd& above	315(56.0)
father’s educational status	no formal education	23(4.1)
	primary education	133(23.7)
	secondary & above	406(72.2)
Occupation of the mother	Government	67(11.9)
	Private	56(10.0)
	House Wife	398(70.8)
	Others (daily laborer & merchant)	41(7.3)
Occupation of the father	Government	100(17.8)
	Private	339(60.3)
	Others (daily laborer& merchant	123(21.9)
Decision-making	mother not involved	35(6.2)
	mother involved	527(93.8)
Family size	2–4	398(70.8)
	5–6	157(27.9)
	7–10	7(1.2)
Household monthly income (Ethiopian Birr)	less than 1500	63(11.2)
	1501–3000	172(30.6)
	3001–4500	57(10.1)
	more than 4500	270(48.0)

### Child and mother health care utilization related characteristics

The majority, (90.4%) of the mothers had a history of antenatal care (ANC) follow-up (≥four times). Five hundred fifty-four, (98.6%) of children were delivered to a health facility. About 94.7% of mothers received postnatal care and 82.6% of children were breastfeeding at the time of the survey **(See** Table 2**)**.

**Table 2:** Child and mother health care characteristics of children 6–23 months, Addis Ababa Ethiopia,2019(*n* = 562)

Variables	Category	Frequency (%)
Antenatal care follow-up	No visit	3(0.5)
	1–3 visits	51(9.1)
	Four and above	508(90.4)
Place of delivery	Home	8(1.4)
	health facility	554(98.6)
Postnatal care follow up	No visit	8(1.4)
	One visit	10(1.8)
	Two visit	12(2.1)
	Three and above	532(94.7)
Current breastfeeding	Yes	464(82.6)
	No	97(17.3)
Additional food started	Less than 6 months	93(16.5)
	At 6 month	420(74.7)
	More than 6 months	47(8.4)

### The practice of minimum acceptable diet

**Fig. 2:**
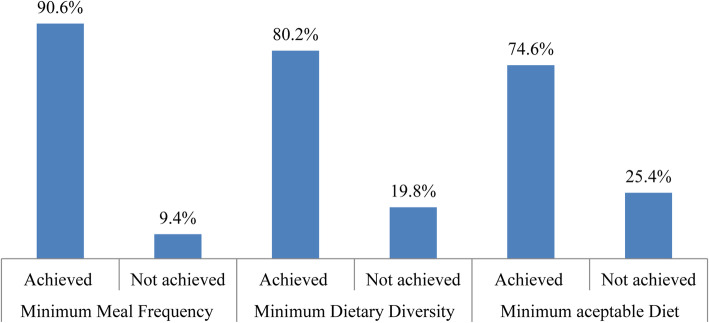
Summary of selected core IYCF indicators (minimum meal frequency, minimum dietary diversity & minimum acceptable diet) among children 6–23 months of age in Addis Ababa, Ethiopia, 2019(*n* = 562)

Nearly three-fourth, 419 (74.6%) of the children fulfilled the recommended minimum acceptable diet criteria. Five hundred six (90.6%) of children received minimum meal frequency and 80.2% of them were received minimum dietary diversity. Four hundred twenty (74.6%) of children started complementary feeding practice in a timely manner, and 55.7% of them had started complementary feeding at an appropriate time (Fig. 2).

### Factors affecting minimum acceptable dietary practices

The results of multivariate analysis showed that the educational status of the father, maternal occupation; postnatal care visit, gender, maternal age, and age of the child were some of the factors associated with a minimally acceptable diet at *P*-value < 0.05.

The age of the mother between 25 and 34 years (AOR = 2.051, 95%CI: 1.267–3.320), being a male child (AOR = 1.585, 95%CI:1.052–2.388), having a postnatal visit of ≥3 (AOR = 2.616, 95%CI:1.120–6.111), age child between 18 and 23 months (AOR = 3.026, 95%CI:1.786–5.128) and fathers education level secondary and above (AOR = 4.789, 95% CI:1.917–11.967) were approximately 2 times, 1.6 times, 2.6 times, 3 times and 4.8 times respectively more likely to have achieved minimal acceptance rate than their counterparts **(See** Table 3**)**.

**Table 3:** Factors associated with minimum acceptable diet practice among children aged 6–23 months, Addis Ababa, Ethiopia,2019 (*n* = 562)

Characteristic	Category	Minimally acceptable diet		COR(95%CI)	AOR(95%CI)
		Achieved	Not achieved		
Age of mother	15–24	67(59.8)	45(40.2)	1	1
	25–34	303(78.5)	83(21.5)	2.452(1.565,3.842***	2.051(1.267,3.320)**
	35–49	49(76.6)	15(23.4)	2.194(1.100,4.378)*	1.793(0.866,3.714)
Gender	Female	198(71.4	79(28.5)	1	1
	Male	221(77.5	64(22.5)	1.378(0.941,2.017)	1.585(1.052,2.388)*
Household monthly income (Ethiopian birr)	< 1500	42(66.7)	21(33.3)	1	1
	1501–3000	116(67.4	56(32.6)	1.036(0.561,1.912)	1.018(0.511,2.027)
	3001–4500	47(82.5)	10(17.5)	2.350(0.994,5.556)	1.891(0.730,4.898)
	> 4500	214(79.3)	56(20.7)	1.911(1.048,3.484)*	1.114(0.509,2.435)
Postnatal care visit	< 3 visit	19(63.3)	11(36.7)	1	1
	≥3 visits	400(75.2)	132(24.8)	1.754(0.814,3.782)	2.616(1.120,6.111)*
Antenatal care visit	< 4 visits	36(66.7)	18(33.3)	1	1
	≥4 visits	383(75.4)	125(24.6)	1.532(0.840,2.794)	1.015(0.514,2.001)
Household food security	No secured	223(71.7)	88(28.3)	1	1
	Secured	196(78.1)	55(21.9)	1.406(0.954,2.072)	1.155(0.740,1.802)
Age of children	12–17 month	138(82.1)	30(17.9)	1	1
	18–23 month	164(78.1)	46(21.9)	2.042(1.310,3.183)**	3.026(1.786,5.128)**
Occupation of mother	Government	60(89.6)	7(10.7	1	1
	Private	46(82.1)	10(17.9)	0.537(0.190,1.518)	0.559(0.191,1.635)
	housewife	279(70.1)	119(229)	0.274(0.121,0.616)**	0.351(0.150,0.819)*
	others	34(82.9)	7(17.1)	0.567(0.183,1752)	0.951(0.289,3.125)
education of mother	No formal education	28(63.6)	16(36.4)	1	1
	Primary	137(67.5)	66(32.5)	1.186(0.600,2.344)	0.869(0.383,1.975)
	2^nd^ary & above	254(80.6)	61(19.4)	2.379(1.212,4.672)*	1.110(4.80,2.852)
Education of the father	No formal education	11(47.8)	12(52.2)	1	1
	Primary	90(67.7)	43(32.3)	2.283(0.933,5.589)	3.597(1.382,9.365**
	2^nd^ary& above	318(78.3)	88(21.7)	3.942(1.682,9.237)**	4.789(1.917,11.967)**
Occupation of the father	Government	75(75.0)	25(25.0)	1	1
	Private	261(77.0)	78(23.0)	1.115(0.664,1.873)	1.810(0.990,3.308)
	Others	83(67.5)	40(32.5)	0.692(0.384,1.247)	1.639(0.778,3.450)
Under-five children	One under five	312(72.1)	121(27.9)	1	1
	Two under five	107(82.9)	22(17.4)	1.886(1.139,3.124)*	1.405(0.743,2.658)
Birth order of index child	First	192(69.1)	86(30.9)	1	1
	≥2	227(79.9)	57(20.1)	1.784(1.213,2.624)**	1.316(0.753,2300)
Number of an individual in the HH	2–4	285(71.6)	113(28.4)	1.009(0.193,5.275)	1.182(0.185,7.555)
	5–6	129(82.2)	28(17.8)	1.843(0.340,9.987)	1.394(0.217,8.944)
	7–10	5(71.4)	2(28.6)	1	1
Mother Decision making	Not involved	20(57.1)	15(42.9)	1	1
	Involved	399(75.7)	128(24.3)	2.338(1.163,4.701)*	1.910(0.875,4.166)

Note: *significant at *p*- value<=0.05and ** significant at *p*-value<=0.001, *** significant at *p*-value<=0.000, *COR* Crude odds ratio, *AOR* Adjusted odds ratio, 1 = reference, *CI* confidence interval

## Discussion

Achieving a minimum acceptable diet among infants and young children of 6–23 months of age is one of the strategies currently used to break the intergenerational cycle of malnutrition. The study findings showed that the proportion of children who received minimum dietary diversity is higher than studies conducted in Addis Ababa (59.9%) [28], Dembecha, North West Ethiopia (9.8%) [10], Gorche District, southern Ethiopia (10.6%) [14]. The difference might be due to the difference in the study area, study period, and educational status of the mothers.

Similarly, the minimum meal frequency of the study was higher than that reported in studies coducted in Ethiopia such as Dabat District (72.2%), Dangla Town (50.4%) and Wolaita Sodo town (68.9%) [13, 15, 29] but it is lower than the study done in Filipino (93.5%) [30]. This discrepancy might be due to the difference in the study setting by which this study was conducted in urban residents and other possibilities might be due to the presence of low attention for child feeding in the previous year and low practice of health care utilization.

In this study much amount of the children who received minimum acceptable diet, which is much higher than findings in Dembecha, North West Ethiopia, Wolaita Sodo town, Northern Shoa (Oromia region), and EDHS 2016 [9, 10, 15, 28, 31]. This is because of the difference in the study period and setting; this study was done in the capital city of Ethiopia (urban setting) and is an institution based which makes the mothers have better educational status and increased awareness to access health service and information on child feeding practice. Other possibilities might be due to an increment of different inter-sectorial and nutritional collaborative programs.

In this study, we have found some factors associated with a minimally acceptable diet. These included; educational status of the father, maternal occupation; history of postnatal care visit, gender, maternal age, and age of the child. Mothers who had a history of more than three postnatal care visits were two times more likely to provide the recommended minimum acceptable diet than their counterparts. This finding is supported by a study done in Northern Shoa, Ethiopia [31]. This is because those mothers who had postnatal care follow-up might have better nutritional advice and counseling provided by health workers during post-natal care visits.

Those children with the age group of 18–23 months had higher odds of having a minimum acceptable diet than those children whose ages were 6–11 months old. This finding is supported by a study done in Afghanistan, rural Ghana, Wolaita Sodo, EDHS 2016 [11, 13, 32, 33]. This might be because mothers may perceive the young child as having the poor ability of the intestine to digest certain foods and the late introduction of complementary feeding might cause the poor feeding interest of the child.

Children born from fathers who had a secondary and above level of education were four times more likely to meet the recommended minimum acceptable diet than those fathers who have no formal education. This study is supported by EDHS, 2016 [33]. This might be because educated fathers were more likely to have information (media exposure) and better understanding for messages and any information about child feeding easily practice.

Children born from mothers who were government employees were 64.9% more likely to receive a minimum acceptable diet than housewives. This finding is in line with EDHS, 2016 [33]. But it is contrary to a study done in Filipino [34]. This might because employed mothers are usually educated and had increased access to the resource, information, and understanding of their social environment than unemployed mothers (housewives).

children who were born from mothers whose ages were in between 25 and 34 years were two times more likely to practice the recommended minimum acceptable diet than the age group of 15–24 years. This finding was similar to a study done in Dabat District, North West Ethiopia [29]. This might be because mothers in this age group are highly productive and had better potential to take care of and feed their children properly.

Finally, male children were more likely to achieve the minimum acceptable diet than female children. The finding was supported with the study done in Ethiopian adolescents [35], West Guji Zone, Oromia, Ethiopia [36] and Sodo zuria District [37]. This might be due to the culture and or traditional perceptions in Ethiopia mostly give high priority to the male baby than females.

## Conclusion

In this study, the practice of minimum acceptable diet in children aged between 6 to 23 months is relatively high in Addis Ababa. Being 18–23 months old child fathers educational level of secondary and above, having more than three postnatal care visits, being a housewife, being male gender, and maternal age between 25 and 34 years were some of the factors associated with the practice of minimum acceptable diet. More efforts should be needed to educate and advocate the benefit of the feeding of recommended minimum acceptable diet for all children to break the intergenerational cycle of malnutrition. On the other hand, it is important FMOH encourage postnatal care service utilization and health professionals who give postnatal care service should also provide education and counseling on the benefit of the feeding of the recommended minimum meal frequency and meal diversity to the child, advise the mother to avoid traditional believes that inhibit the practice of child feeding. Additionally, it is important to do on the gender equality of feeding practice of the mother. A strong study design (cohort) should be recommended.

### Limitations of the study

Being a health facility-based study particularly governmental health facilities ignored the entire community and those who had served in private health facilities, might overestimate the finding. In addition to this, as the study considered only 24 h recall dietary method, it might not accurately reflect participants’ past feeding dietary habits. Moreover, there might be a recall bias, and being a self-reported study might not give the exact figure of the minimum dietary diversity practice (social desirability bias).

## Data Availability

The full data set and other materials about this study can be obtained from the corresponding author on reasonable request.

## Abbreviations

ANC: Antenatal Care
CSA: Central Statistical Agency
EDHS: Ethiopian Demographic Health Survey
EPI: Expanded Program on Immunization
MDD: Minimum Dietary Diversity
MMF: Minimum Meal Frequency
MOH: Ministry of Health
MCH: Maternal and Child Health care
SPSS: Statistical Package for Social Sciences
WHO: World Health Organization
